# Detection and Speed Estimation of Moving Target Based on Phase Compensation and Coherent Accumulation Using Fractional Fourier Transform

**DOI:** 10.3390/s20051410

**Published:** 2020-03-04

**Authors:** Jian Yang, Xinxin Liu, Bo Yang, Jian Lu, Guisheng Liao

**Affiliations:** 1 School of Electronic Engineering, Xidian University, Xi’an 710071, China; liaogs@xidian.edu.cn; 2 School of Engineering, Rocket Force University of Engineering, Xi’an 710025, China; neuqliuxinxin@163.com (X.L.); yangbo8093@sina.com (B.Y.); lujiansc@tom.com (J.L.)

**Keywords:** target detection, phase compensation, coherent accumulation, Fourier transform

## Abstract

As unmanned aerial vehicles and other small, low-flying, and low-speed aircrafts are being extensively used, studies on their detection are being extensively conducted in radar application research. However, weak echoes, low Doppler frequencies, and target echoes mixed with ground clutter can considerably degrade the detection performance. Therefore, specific methods for the detection of such targets should be devised. We propose herein a phase compensation and coherent accumulation algorithm based on the fractional Fourier transform (FRFT) for detection and speed estimation of this type of target. First, the energy of the target echo is converged using the FRFT. Next, the phase between the peaks of the target echo is analyzed. Phase compensation and coherent accumulation determined from the expected target speed in the fractional domain eliminate ground clutter and further improve the signal-to-interference-plus-noise ratio. Finally, constant false alarm rate detection is used to identify the target, for which radial speed can be estimated directly according to the peak coordinates. The validity of the algorithm is verified via data simulation and application to real data.

## 1. Introduction

The wide application of low-flying, small, and slow aircrafts in fields such as mapping, military operations, aerial photography, agricultural production, environmental monitoring, and search-and-rescue, has contributed to the advancement of technological solutions in recent years. However, more and more illegal unmanned aerial vehicles (UAVs) threaten the security of airports, ports, densely populated areas, and military facilities. Therefore, the detection of low-flying, small, and slow targets has attracted much research attention [1,2,3]. However, the characteristics of such targets results in radar echoes that have a small radar cross-section (RCS), low Doppler frequency, and susceptibility to ground clutter-factors that hinder accurate and timely detection [4,5]. Currently, research on the effective detection of low-flying, small, and slow targets is scarce; thus, we aim to devise a method to detect such targets and estimate their speeds.

Various methods have been proposed to detect small targets. In [6,7], the Radon–Fourier transform, and its generalized variant, were proposed to realize long-time coherent accumulation for radar detection of targets with arbitrary parameterized motion. In [8], a rapid estimation method based on the adjacent cross-correlation function was devised via modeling the slant range of the target as a polynomial function over multiple motion parameters. In [9], a Radon-fractional ambiguity function was proposed to compensate for the range and Doppler migrations simultaneously. Using a long-time instantaneous autocorrelation function and the rotation of the time–frequency plane, the observation values of the target were matched and accumulated as a peak in the Radon-fractional ambiguity function domain. In [10], fast coherent accumulation based on the time reversal transform, second-order keystone transform, and Lv’s distribution was proposed to perform coherent accumulation for detecting a maneuvering target with high-order range migration. These algorithms mainly aim to achieve long-time coherent accumulation by correcting range migration and Doppler frequency migration and enhancing the signal-to-interference ratio of the target echo for detecting small and weak-signal targets. However, given the low speed and mobility of slow and low-flying targets, the range migration and Doppler frequency migration of targets are not clearly reflected in low-resolution radar systems. Moreover, the abovementioned detection algorithms are time-consuming, thus undermining the detection efficiency. In [11], a low-cost acoustic array was designed to locate and track small UAVs in the far-field via algorithms for array calibration and beamforming, however the obtained detection performance was low. Based on the multi-living agent information system theory, an architecture for detection of low-flying, small, and slow targets was proposed in [12]. In [13], a passive detection algorithm aimed to autonomously detection and characterization of drones using radio-frequency wireless signals was developed. This method mainly enabled interception of the communication signal of a UAV via passive listening, consequently providing functionality in a short detection distance. In [14], a distributed frequency-modulated continuous-wave radar system based on fiber-optic links was designed to detect a small drone within a 500 m range; however, this distance proved to be insufficient. In [15], the multiple frequency shift keying transmit signal was developed to measure target range and radial velocity separately and simultaneously, which was a combination of the classical chirp sequence and an additional frequency shift keying or frequency modulation component. However, the frequency modulated continuous waveform signals and the bandwidth over 100MHz in target detection limited the application of other processing algorithms, and the detection distance of the system was only hundreds of meters. In [16], a two-dimensional (2D) fast Fourier transform was used to detect a UAV in the presence of dense architectural clutter via a linear frequency modulated (LFM) continuous wave radar. The technology of “Strech” [17] had been applied in [15,16], which effectively reduced the requirements for A/D sampling rate and subsequent digital signal processing speed. However, it increased the complexity of hardware mixing, and greatly reduced the detection distance of range. Chen et al. [18]. proposed a long-time coherent integration (LTCI) method for radar maneuvering targets, especially for low-observable unmanned aerial vehicle targets. In order to the improve signal-to-noise ratio, the problem of range migration had to be considered and compensated. The computational cost was relatively large and the moving target detection (MTD) performance of this algorithm in different clutter environments had to be further verified. In addition, the research on the fractional Fourier transform (FRFT) has made some important achievements in recent years. Guan et al. [19] proposed an adaptive line enhancer (ALE) in the FRFT domain to suppress sea clutter and improve the signal-to-clutter ratio, which provided less error and faster convergence. However, the performance of the algorithm was related to the model of sea clutter, and a variable step size ALE needs to be further studied. Liu et al. [20] proposed a sparse discrete FRFT algorithm to reduce the computational complexity when dealing with large data sets that were sparsely represented in the fractional Fourier domain. Moreover, they further combined the radar-based modality and FRFT to achieve higher signal energy concentration and yield improved fall detection in low signal-to-noise ratio scenarios [21]. Li et al. [22] proposed a new technique relying on a hybrid coherent/non-coherent integration of the received signal in the FRFT domain to detect the moving targets for a space-based passive radar, and the effectiveness was proved by the reliable detection of low observable targets. In [23], a method known as Radon-FRFT was proposed and investigated to improve the radar detection ability of a weak maneuvering target by the long-time coherent integration technique. However, the timeliness of the target detection is insufficient.

Generally, radars adopt large bandwidth signals and long-time accumulation to distinguish targets from strong background noise. Based on existing radar systems, we use the transmission of narrowband detection signals to detect low-flying, small, and slow targets in a short time. The fast detection and parameter estimation for such targets is achieved by optimizing the corresponding signal processing algorithms.

The remainder of this paper is organized as follows. Section 2 describes the signal model. Section 3 introduces the detection and parameter estimation algorithm of low-flying, small, and slow targets based on fractional-domain phase compensation and coherent accumulation. This section includes the definition of the fractional Fourier transform (FRFT), target echo transform, phase compensation, coherent accumulation, and overall algorithm. Section 4 presents simulation results of the algorithm that verify its performance. In Section 5, experiments on the measured data further verify the effectiveness of the algorithm and its superior performance compared to an existing algorithm. Finally, in Section 6, we provide concluding remarks.

## 2. Signal Model

The detection of low-flying, small, and slow targets using radars results in echoes that primarily involve three aspects—ground clutter, target, and system noise—with the ground clutter signal usually being the strongest. During detection, we assume that the echo contains information from a low-flying, small, and slow target and *K* ground objects as follows:

(1) $$S_{r}(\tau ,t_{m})=s_{IF_{0}}(\tau ,t_{m})+\sum_{k=1}^{K}s_{IF_{k}}(\tau ,t_{m})+n(\tau ),$$

where $t_{m}=mT_{pri}\;(m=0,1,2,\ldots ,M)$ is the slow time, with $T_{pri}$ denoting the pulse repetition interval, and M is the number of coherent integrated pulses; $\tau =t-mT_{pri}$ is the fast time, i.e., the intra-pulse time, with t denoting the total time; $s_{IF_{0}}(\tau )$ is the echo of the target, $s_{IF_{k}}(\tau ,t_{m})$ is the echo of the *k*-th fixed object, and n(τ) represents the system noise following a Gaussian distribution with zero mean and variance $\delta^{2}$.

If the radar transmits an LFM signal during target detection, the signal can be expressed as

(2) $$s_{t}(t)=rect\left(\frac{t}{T_{p}}\right)\exp\left[j2\pi \left(f_{c}t+\frac{1}{2}\kappa t^{2}\right)\right]$$

with

$$rect(x)=\{\begin{array}{c} 1,\;\left|x\right|\leq \frac{1}{2}\\ 0,\;\left|x\right|>\frac{1}{2} \end{array}$$

where $T_{p}$ is the pulse width, $f_{c}$ is the carrier frequency, $\kappa =B/T_{p}$ is the frequency modulation rate of the LFM signal, with bandwidth B. The *k*-th target echo received by the radar can be expressed as

(3) $$s_{k}(\tau ,t_{m})=\delta_{k}rect\left(\frac{\tau -\tau_{k}}{T_{p}}\right)\exp\left\{j2\pi \left[f_{c}(\tau -\tau_{k})+\frac{1}{2}\kappa {\left(\tau -\tau_{k}\right)}^{2}\right]\right\},\;\;0\leq k\leq M$$

where $\delta_{k}$ is the backscatter coefficient of the target; and $\tau_{k}=2r_{k}(t_{m})/c$ is the delay, where c is the speed of light, and $r_{k}(t_{m})$ is the line-of-sight distance between the radar and target, which is a function of $t_{m}$. Therefore, the radar echo depends on time $t_{m}$ and τ, which represent the slow-time and the fast-time, respectively.

During signal processing, a rectangular coordinate system with the radar position as the origin is established. Assuming that a low-flying, small, and slow target is moving in the direction of the radar, its velocity can be decomposed into radial and tangential velocity components, of which only the former has an effect on the range change. If the slant distance of the target is $r_{0}$ at time t=0, the distance of the target with respect to time can be obtained using the following Taylor series expansion:

(4) $$r_{s}(t_{m})=r_{0}-vt_{m}-\frac{1}{2}v^{\prime }t_{m}^{2}-\ldots ,\;\;\;t_{m}\in \left[-\frac{T_{m}}{2},\frac{T_{m}}{2}\right],$$

where v is the speed of the target, $v^{\prime }$ is the first derivative of v, and $T_{m}$ is the coherent accumulation time. For low-flying, small, and slow targets, the maneuverability is low. Thus, in a short coherent accumulation time $T_{m}$, the acceleration of the target can be considered to be zero (i.e., $v^{\prime }=0$), and the distance of the target can be expressed as

(5) $$r_{s}(t_{m})=r_{0}-vt_{m}.$$

By demodulating the carrier frequency signal, the zero intermediate-frequency signal can be expressed as

(6) $$\begin{array}{l} s_{IF_{0}}(\tau ,t_{m})=s_{0}(\tau ,t_{m})\cdot \exp\left(-j4\pi f_{c}\frac{r_{0}-vt_{m}}{c}\right)\\ =\delta_{0}\exp\left(-j4\pi f_{c}\frac{r_{0}-vt_{m}}{c}\right)\cdot rect\left[\frac{\tau -2(r_{0}-vt_{m})/c}{T_{p}}\right]\exp\left\{j\pi \kappa {\left[\frac{\tau -2(r_{0}-vt_{m})}{c}\right]}^{2}\right\} \end{array}$$

Thus, for a low-flying, small, and slow target, after the radar echo is processed upon carrier frequency removal, its echo remains as an LFM signal with the frequency modulated rate κ. Likewise, for the ground object, regardless of whether it is a slow moving or static ground object, the echo remains as an LFM signal with the same frequency modulated rate κ.

Therefore, low-flying, small, and slow targets possess signal characteristics of slow movement and weak radar echo with strong ground object interference. For detecting the weak signal from the target, pulse-to-pulse echo accumulation can be adopted to enhance both the signal-to-interference-plus-noise ratio (SINR) of the received signal and detection probability. In case of the proposed algorithm, the low speed characteristic of the targets increases the number of pulses that can be accumulated, thereby improving the efficiency of echo signal accumulation, and restricts the processing range of compensation for target speed, further improving the efficiency of whole signal processing. The limited maneuverability of the target allows simplifying its motion model in a short period as radial uniform motion, and the echo can be expressed as an LFM signal with the same frequency modulated rate κ as the transmission signal, while the phase differences between the continuous target echoes are closely related to the radial velocity of the target and the pulse repetition interval of the radar transmitted signals. For a low altitude, the target echo is easily interfered by ground clutter. Moreover, the echo remains an LFM signal with frequency modulated rate κ, but no (or only a very small) phase change occurs between the continuous pulse echoes. Given the low speed of low-flying, small, and slow targets, their echoes can be treated considering a limited speed range to avoid unnecessary processing.

## 3. Proposed Algorithm for Target Detection and Speed Estimation

By analyzing the radar echo of a low-flying, small, and slow target, we determined that the echo is an LFM signal with the same modulation frequency as that of the transmitted signal, as verified by the FRFT of the signal, which exhibits a suitable energy convergence effect under the appropriate transformation order. Therefore, for target detection, we analyze the peak phase relationship of the target echo in the fractional domain and propose a method for MTD and speed estimation by phase compensation and coherent accumulation in the fractional Fourier domain.

### 3.1. Fractional Fourier Transform

The FRFT is a linear time–frequency analysis method proposed by Namias in 1980 [24]. It represents a signal from an angle with anticlockwise rotation from the time axis α to axis u (i.e., the FRFT domain). The transformed signal can contain both time- and frequency-domain information. The *p*-order FRFT of time-domain signal x(t) can be expressed as in [25,26]:

(7) $$X_{\alpha }(u)=F^{p}[x(t)]=\int_{-\infty }^{+\infty }x(t)K_{p}(t,u)dt,$$

where $K_{p}(t,u)$ is the kernel function of the transform, which is given by

(8) $$K_{p}(t,u)=\left\{ \begin{array}{ll} \sqrt{\frac{1-j\cot\alpha }{2\pi }}\exp\left[j\pi \left(\frac{u^{2}+t^{2}}{2}\cot\alpha -ut\csc\alpha \right)\right], & \alpha \neq n\pi \\ \delta (t-u), & \alpha =2n\pi \\ \delta (t+u), & \alpha =(2n+1)\pi \; \end{array}\right.,$$

where $\alpha =p\frac{\pi }{2}$ represents the rotation angle in the time–frequency domain.

The FRFT exhibits many advantageous properties, such as linearity, reversibility, time shift, and frequency shifting [27]. The linearity is given by

(9) $$F^{p}[\sum_{k}a_{k}f_{k}(t)]=\sum_{k}a_{k}F^{p}[f_{k}(t)],$$

where k represents the number of functions being added. Reversibility is expressed as

(10) $${\left(F^{p}\right)}^{-1}=F^{-p}.$$

The transformation under time shift $\tau_{0}$ is expressed as

(11) $$F^{p}[x(t-\tau_{0})]=\exp(j\pi \tau_{0}^{2}\sin\alpha \cos\alpha -j2\pi u\tau_{0}\sin\alpha )F^{p}(u-\tau_{0}\cos\alpha ),$$

and that under frequency shift $f_{0}$ is expressed as

(12) $$F^{p}[x(t)\exp(j2\pi f_{0}t)]=\exp(-j\pi f_{0}^{2}\sin\alpha \cos\alpha +j2\pi uf_{0}\cos\alpha )F^{p}(u-f_{0}\sin\alpha ).$$

### 3.2. FRFT of Target Echo

The transmitting signal in Equation (2) after carrier frequency removal can be expressed as

(13) $$s_{0}(t)=rect\left(\frac{t}{T_{p}}\right)\exp(j\pi \kappa t^{2}),$$

When $\alpha -\arctan(\kappa )\neq \frac{2n+1}{2}\pi$ for integer *n*, the FRFT of the LFM signal $s_{0}(t)$ can be written as in [28]:

(14) $$\begin{array}{l} F^{p}[s_{0}(t)]=\int_{-\infty }^{+\infty }s_{0}(t)K_{p}(t,u)dt\\ =\sqrt{\frac{1+j\tan\alpha }{1+\kappa \tan\alpha }}\exp\left[\frac{j\pi \alpha^{2}(\kappa -\tan\alpha )}{1+\kappa \tan\alpha }\right] \end{array}.$$

When $\alpha -\arctan(\kappa )=\frac{2n+1}{2}\pi$, the transform exhibits energy convergence in the u domain:

(15) $$F^{p_{0}}[s_{0}(t)]=\sqrt{1-j\cot\alpha_{0}}\exp(j\pi u^{2}\cot\alpha_{0})\frac{\sin(\pi u\csc\alpha_{0}T_{o})}{\pi u\csc\alpha_{0}},$$

where $T_{0}$ is the observation time, $\alpha_{0}=-\arctan(1/\kappa )$ is the optimal rotation angle, and $p_{0}=\frac{2}{\pi }\alpha_{0}$ is the optimal transformation order. In discrete time, when the frequency modulation rate κ of the LFM signal is known, the optimal rotation angle of the transform can be calculated as in [29,30]:

(16) $$\alpha_{o}=\frac{2}{\pi }\arctan\left(\frac{f_{s}^{2}/N_{s}}{2\kappa }\right),$$

where $f_{s}$ is the sampling frequency and $N_{s}$ is the length of the data to be processed.

According to Equation (15), when u=0, the peak of $F^{p_{0}}[s_{0}(t)]$ in the u domain is given by

(17) $$F^{p_{0}}{\left[s_{0}(t)\right]}_{\max}=\sqrt{1-j\cot\alpha_{0}}T_{o}.$$

From Equations (6) and (11), the target echo $s_{IF_{k}}(t,t_{m})$ can be obtained by multiplying the transmitted signal $s_{0}(t)$ by an amplitude phase and a time delay change:

(18) $$s_{IF_{k}}(t,t_{m})=\delta_{k}\exp(-j2\pi f_{c}\tau_{k})\cdot s_{0}(t-\tau_{k}).$$

Then, according to Equations (9), (11) and (18), the FRFT of the echo for a low-flying, small, and slow target is given by

(19) $$\begin{array}{l} F^{p_{0}}[s_{IF_{0}}(t,t_{m})]=\delta_{0}\exp(-j2\pi f_{c}\tau_{0})\cdot F^{p_{0}}[s_{0}(t-\tau_{0})]\\ =\delta_{0}\exp(-j2\pi f_{c}\tau_{0})\cdot \exp(j\pi \tau_{0}^{2}\sin\alpha_{0}\cos\alpha_{0}-j2\pi u\tau_{0}\sin\alpha_{0})F^{p_{0}}[s_{0}(t)](u-\tau_{0}\cos\alpha_{0}) \end{array},$$

where $F^{p_{0}}[s_{0}(t-\tau_{k})]$ is the FRFT of a shifted function $s_{0}(t-\tau_{k})$, and $F^{p_{0}}[s_{0}(t)](u-\tau_{0}\cos\alpha_{0})$ is a shifted function of $F^{p_{0}}[s_{0}(t)](u)$ in the fractional domain.

For the echo of a moving target, $\tau_{0}=2(r_{0}-vt_{m})/c$ constantly changes overtime, whereas for the echo of a fixed ground object *k*, the time delay remains unchanged as $\tau =\tau_{k}=2r_{k}/c$, where $r_{k}$ is the line-of-sight distance between the *k*-th fixed ground object and the radar. Hence, the amplitude and phase of the peak value in the fractional domain remain unchanged within a certain period, and the FRFT of its echo can be expressed as

(20) $$\begin{array}{l} F^{p_{0}}[s_{IF_{k}}(t,t_{m})]=\delta_{k}\exp(-j2\pi f_{c}\tau_{k})\cdot F^{p_{0}}[s_{0}(t-\tau_{k})]\\ =\delta_{k}\exp(-j2\pi f_{c}\tau_{k})\cdot \exp(j\pi \tau_{k}^{2}\sin\alpha_{0}\cos\alpha_{0}-j2\pi u\tau_{k}\sin\alpha_{0})F^{p_{0}}[s_{0}(t)](u-\tau_{k}\cos\alpha_{0}) \end{array},$$

where $\delta_{k}$ is the backscatter coefficient of the *k*-th object and $\tau_{k}$ is the echo delay time determined by the slant distance between the *k*-th object and the radar.

### 3.3. Phase Compensation and Coherent Accumulation

For the echo of a low-flying, small, and slow target, according to Equation (19), when $u=\tau_{0}\cos\alpha_{0}$, $F^{p_{0}}[s_{IF_{0}}(t,t_{m})]$ can be taken as the maximum value:

(21) $$F^{p_{0}}{\left(s_{IF_{0}}\right)}_{\max}=\delta_{r_{0}}\exp(-j2\pi f_{c}\tau_{0})F^{p_{0}}{\left[s_{0}(t)\right]}_{\max}=\delta_{r_{0}}\exp\left(j4\pi f_{c}\frac{v}{c}t_{m}\right)\exp\left(-j4\pi f_{c}\frac{r_{0}}{c}\right)F^{p_{0}}{\left[s_{0}(t)\right]}_{\max}$$

where $\delta_{r_{0}}=\delta_{0}\cdot \exp(j\pi \tau_{0}^{2}\sin\alpha_{0}\cos\alpha_{0}-j2\pi u\tau_{0}\sin\alpha_{0})$. For a ground clutter signal, according to Equation (20), when $u=\tau_{k}\cos\alpha_{0}$, $F^{p_{0}}[s_{IF_{k}}(t,t_{m})]$ can be expressed as

(22) $$F^{p_{0}}{\left(s_{IF_{k}}\right)}_{\max}=\delta_{r_{k}}\exp(-j2\pi f_{c}\tau_{k})F^{p_{0}}{\left[s_{0}(t)\right]}_{\max},$$

where $\delta_{r_{k}}=\delta_{k}\cdot \exp(j\pi \tau_{k}^{2}\sin\alpha_{0}\cos\alpha_{0}-j2\pi u\tau_{k}\sin\alpha_{0})$. From Equation (21), the peak phase of the continuous pulse echo in the fractional domain changes according to the echo of the moving target. From Equation (22), the peak phase and amplitude of the continuous pulse echo in the fractional domain remain relatively constant for the signal of a fixed ground object, and their peaks are determined by the distance and rotation angle. Subsequently, we can derive the following phase compensation factor:

(23) $$\varphi (v)=\exp\left(-j4\pi f_{c}\frac{v}{c}t_{m}\right)=\exp\left(-j4\pi f_{c}m\frac{v}{c}T_{pri}\right),$$

where m is the number of pulses, and $T_{pri}$ is the pulse repetition interval. To ensure that the target motion does not cause range migration, the peak of the continuous pulse echo of the moving target is expressed as

(24) $$F_{s_{0}}^{p_{0}}=\varphi (v)\cdot F^{p_{0}}{\left(s_{IF}\right)}_{\max}=\delta_{r_{0}}\exp\left(-j4\pi f_{c}\frac{r_{0}}{c}\right)F^{p_{0}}{\left[s_{0}(t)\right]}_{\max}.$$

After phase compensation of the FRFT of the continuous pulse echo from a fixed object, the peak is given by

(25) $$F_{s_{k}}^{p_{0}}=\varphi (v)\cdot F^{p_{0}}{\left(s_{IF_{1}}\right)}_{\max}=\delta_{r_{k}}\exp(-j2\pi f_{c}\tau_{k})\exp\left(-j4\pi f_{c}\frac{v}{c}t_{m}\right)F^{p_{0}}{\left[s_{0}(t)\right]}_{\max}.$$

After coherent accumulation of Equations (24) and (25), we respectively obtain

(26) $$C_{s_{0}}=\frac{1}{M}\sum_{m=1}^{M}F^{p_{0}}[s_{IF_{0}}(t,t_{m})]\approx \delta_{r_{0}}\exp\left(-j4\pi f_{c}\frac{r_{0}}{c}\right)F^{p_{0}}{\left[s_{0}(t)\right]}_{\max},$$

(27) $$\begin{array}{l} C_{s_{k}}=\frac{1}{M}\sum_{m=0}^{M-1}F^{p_{0}}[s_{IF_{k}}(t,t_{m})]\approx \delta_{k}\exp(-j2\pi f_{c}\tau_{k})F^{p_{0}}{\left[s_{0}(t)\right]}_{\max}\sum_{m=0}^{M-1}\exp(-j4\pi f_{c}m\frac{v}{c}T_{pri})\\ =\frac{1}{M}\delta_{k}\exp(-j2\pi f_{c}\tau_{k})F^{p_{0}}{\left[s_{0}(t)\right]}_{\max}\cdot \frac{1-\exp(-j4\pi f_{c}M\frac{v}{c}T_{pri})}{1-\exp(-j4\pi f_{c}\frac{v}{c}T_{pri})} \end{array},$$

where M is the number of coherent cumulative pulses.

Let $1-\exp(-j4\pi f_{c}M\frac{v}{c}T_{pri})=0$ to obtain the optimal number of coherent cumulative pulses,

$$4\pi f_{c}M\frac{v}{c}T_{pri}=2n\pi$$

with n being an integer. Then,

(28) $$M=round\left(\frac{nc}{2f_{c}vT_{pri}}\right),$$

where round(·) represents the rounding operation.

For coherent processing of the target echo, we assume that the target displacement does not exceed half of the range resolution unit of radar within the coherent processing time of the *M* continuous pulse echoes:

(29) $$v\cdot T_{m}=MT_{pri}v\leq \frac{\Delta r}{2}=\frac{c}{4B},$$

where $T_{m}$ denotes the maximum movement time of the target without range cell migration. The number of pulses that can be included in incoherent processing must meet the following conditions:

(30) $$N=\left\lfloor \frac{T_{m}}{T_{pri}}\right\rfloor \leq \left\lfloor \frac{c}{4Bv}\right\rfloor ,$$

where ⌊·⌋ represents the rounding down operation.

Therefore, according to Equations (28) and (30), the number M of pulse echoes included in coherent accumulation must meet the following constraints:

(31) $$M=round\left(\frac{nc}{2f_{c}vT_{pri}}\right),\;M\leq \left\lfloor \frac{c}{4Bv}\right\rfloor$$

By selecting the appropriate number of coherent cumulative pulses *m*, the echo of the ground object can approach zero in the fractional domain (i.e., $C_{s_{k}}=0$). Note that the FRFT of a Gaussian function contains complex variables [31], and during coherent processing, the noise converges to zero in the fractional Fourier domain. Therefore, the transformation in Equation (1) after phase compensation and coherent accumulation can be expressed as

(32) $$\begin{array}{l} \frac{1}{M}\sum_{m=0}^{M-1}F^{p_{0}}[S_{r}(t,t_{m})]=\frac{1}{M}\sum_{m=0}^{M-1}F^{p_{0}}[s_{IF_{0}}(t,t_{m})]+\frac{1}{M}\sum_{m=0}^{M-1}F^{p_{0}}[\sum_{k=1}^{K}s_{IF_{k}}(t,t_{m})]+\frac{1}{M}\sum_{m=0}^{M-1}F^{p_{0}}[s_{n}(t,t_{m})]\\ \approx C_{s_{0}}=\delta_{r}\exp\left(-j4\pi f_{c}\frac{r_{0}}{c}\right)F^{p_{0}}{\left[s_{0}(t)\right]}_{\max} \end{array}$$

In the *u–v* 2D graph of coherent accumulation results, we identify the effective moving target by constant false alarm rate (CFAR) detection and estimate the radial velocity of the target by obtaining the velocity coordinate corresponding to its peak. The realization of CFAR detection is based on the assumption that the data after phase compensation and coherent accumulation in the FRFT domain obey Gaussian distribution. The detection threshold can be obtained as

(33) $$T_{h}=\mu +P_{G}^{-1}(p_{F})\delta ,$$

where $P_{G}(S)=1-\Phi (S)$ with Φ(·) denoting the Standard Normal Distribution Function, $P_{G}^{-1}(\cdot )$ denotes the inverse function of the function $P_{G}(S)$, $p_{F}$ denotes the false-alarm rate, μ and δ denote the mean and standard deviant of the *u–v* 2D data in the FRFT domain, respectively. If the data are greater than $T_{h}$, the peaks among them can be searched and considered as the effective moving targets.

### 3.4. Target Detection and Speed Estimation Algorithm

**Figure 1:**
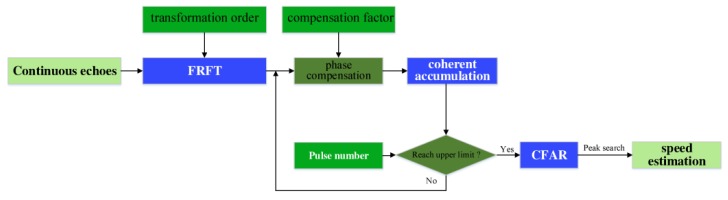
Flowchart of proposed algorithm.

We propose the detection method illustrated in Figure 1 for low-flying, small, and slow targets in the fractional domain for coherent accumulation. The algorithm proceeds as follows.

(1) Receive *m* continuous echoes $s_{r}(t,t_{m})$, 0≤m≤M−1, and obtain $s_{IF}(t,t_{m})$ by down-conversion.
(2) According to Equation (16), calculate the optimal rotation angle $\alpha_{0}$ and apply the $p_{0}$ order FRFT to the M echoes.
(3) Set the range of target speed $v\in [-v_{\max},+v_{\max}]$, where $v_{\max}$ is predefined to represent the maximum speed of the targets. Calculate the number of accumulated pulses under speed v according to Equation (31) and compensation factor φ(v) according to Equation (23).
(4) Compensate for the FRFT results via factor φ(v) and then apply coherent accumulation according to Equation (32).
(5) Detect the moving target using CFAR detection and peak search. Estimate the speed of the target according to the speed coordinate in the *u*–*v* diagram.

The proposed algorithm is compared with the conventional MTD in terms of computational complexity. Because both the algorithms employ the CFAR detection method, the comparison of computational complexity is mainly based on the data processing in the preceding steps. For the proposed algorithm, the main complexity lies in the FRFT of all received echoes, phase compensation and coherent accumulation. The computational complexity of the former is $O(MN_{s}\log N_{s})$, and the latter can be implemented at a complexity cost of $O(MN_{v})$, where $N_{v}$ is the number of sampling points in the target speed range. As $N_{v}$ is generally smaller than $N_{s}$, the algorithmic complexity of the proposed algorithm is $O(MN_{s}\log N_{s})$. For the general MTD method, the main complexity lies in the pulse compression and coherent accumulation. The pulse compression requires a computational load of $O(MN_{s}\log N_{s})$, and the coherent accumulation costs corresponding to $O(N_{s}M\log M)$. Thus, the algorithmic complexity of MTD is $O(\max(MN_{s}\log N_{s},N_{s}M\log M))$. Consequently, from a computational complexity point of view, the proposed algorithm is superior to that of the general MTD method.

## 4. Numerical Simulations

For simulation, we set the radar parameters as follows: Center frequency $f_{c}=1500\;\mathrm{MHz}$, pulse width $T_{p}=10\;\mu s$, bandwidth B=5 MHz, pulse repetition interval $t_{pri}=200\;\mu s$, sampling frequency $f_{s}=10\;\mathrm{MHz}$, and pulse echo sampling length $N_{s}=2000$. We considered two low-flying, small, and slow aerial vehicles and two fixed objects in the radar detection range, at distances of 26.8, 21.1, 13.4, and 25.5 km, respectively, with speeds of $v_{1}=15.36\;m/s$ and $v_{2}=-23.58\;m/s$, and a signal-to-noise ratio of the expected echo of 10 dB for the vehicles and 25 dB for the fixed objects. According to these conditions, the radar range resolution was 30 m, and the target movement did not induce range migration within *M* = 3125 pulses. Therefore, during coherent accumulation, the number of continuous pulse echoes per speed value should be limited to *M*.

**Figure 2:**
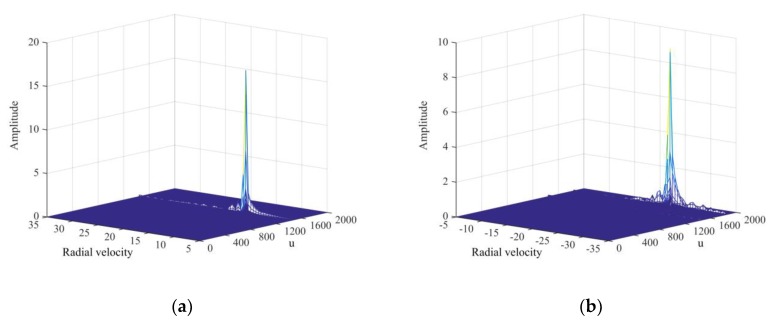
Simulation results of phase compensation and coherent accumulation: (**a**) Target detection with positive velocity ${\tilde{v}}_{1}=15.5\;m/s$; (**b**) target detection with negative velocity ${\tilde{v}}_{2}=-23.5\;m/s$.

During the simulation, we generated 2048 continuous pulse echoes, and phase compensation and coherent accumulation were applied after the FRFT to form a 2D *u*–*v* diagram as shown in the Figure 2. The coordinate velocity data pair was generated via simulation. Despite the fact that positive and negative velocities are processed with the same method, two *u*–*v* 2D graphs distinguishing positive and negative speed are shown to demonstrate the processing results more clearly. As shown in Figure 2, peaks exist in the positive and negative velocity regions with their coordinates of (15.5, 1442) and (−23.5, 1840), and the speed estimates are ${\tilde{v}}_{1}=15.5\;m/s$ and ${\tilde{v}}_{2}=-23.5\;m/s$, respectively. The real values verify the high accuracy of the speed estimates.

**Figure 3:**
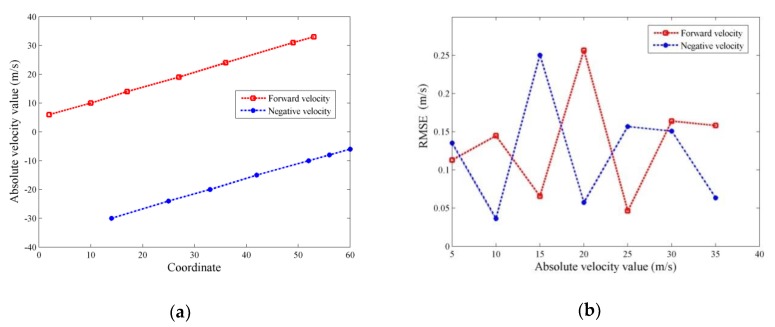
Target speed estimation: (**a**) Relationship between coordinate and speed; (**b**) speed estimation error.

According to the characteristics of the proposed algorithm, there is a linear relationship in theory between the velocities of the moving target and coordinates of *u* axis. In order to verify the linear relationship in the *u–v* 2D graph, several speed values within the range of the target speed were randomly selected to be the simulation parameters, then the proposed algorithm was applied to obtain the estimated velocity of the moving target. Via acquiring multiple estimated velocity-coordinate value pairs, on the one hand, the corresponding relationship between them can be verified and, on the other hand, the effectiveness of the proposed algorithm for detecting the moving target can be proven. In the simulation results, as shown in Figure 3a, there is a linear relationship between the estimated values of speed and the coordinate values. The linear relationship of the estimated positive and negative velocities with the corresponding coordinates can be expressed as $v_{+}=c_{11}n_{+}+c_{12}$ and $v_{-}=c_{21}n_{-}+c_{22}$, respectively, where $v_{+}$ denotes the estimation of positive velocity, $v_{-}$ denotes the estimation of negative velocity, $c_{11}$, $c_{12}$, $c_{21}$, and $c_{22}$ are the coefficients of linear fitting, $n_{+}$ and $n_{-}$ denote the coordinate values in the *u–v* 2D graphs. Moreover, these coefficients, $c_{11}=0.5322$, $c_{12}=4.8227$, $c_{21}=0.5214$, and $c_{22}=-37.1489$, can be obtained via least squares estimation with the estimated velocity-coordinate value pairs. In simulation, we obtained the root-mean-square error of velocity estimation as follows.

(34) $$V_{RMSE}=\sqrt{\frac{1}{N_{z}}\sum_{n=1}^{N_{z}}{\left({\tilde{v}}_{n}-v_{n}\right)}^{2}},$$

**Figure 4:**
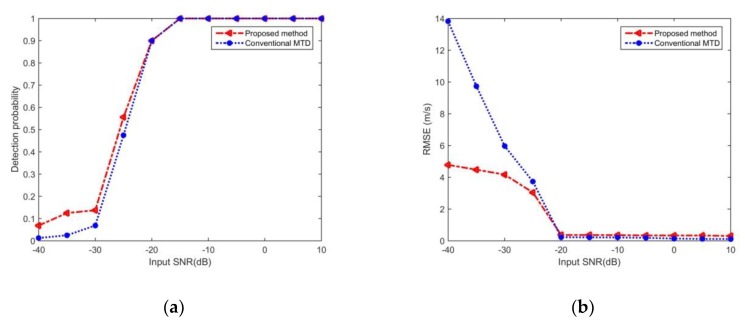
Performance comparison: (**a**) Detection probability over input SNR; (**b**) Speed estimation error over input SNR.

To further verify the performance of the proposed algorithm, the proposed method is compared with the general MTD including detection probability over SNR and velocity estimation accuracy over SNR. The radar parameters are the same as the former simulation trials. In the simulating scenarios, there are two moving targets with positive speed and negative speed, respectively. Moreover, their velocities are changed after each simulation, which absolute values obey the uniform distribution in the range of [5 m/s,35 m/s], but kept fixed from echo to echo during each simulation, and 1024 continuous echoes are utilized to detect the moving targets in each simulation. For the proposed method, the velocity interval during phase compensation and the false alarm rate in CFAR detection are set as 0.1 m/s and 0.0001, respectively. For each simulation, 200 Monte-Carlo runs are performed. The results of simulation comparison are shown in Figure 4. The detection probability over SNR is shown in Figure 4a, and RMSE of the velocity estimation over SNR is demonstrated in Figure 4b. It can be observed from the Figure 4a that the detection probability of the proposed method is higher than that of the general MTD when SNR is lower than −20 dB, and their performance is equivalent on other SNRs. Figure 4b demonstrates that the speed estimation accuracy of the proposed method is superior to that of the general MTD when SNR is lower than −20 dB, and the speed estimation errors are slightly greater than the general MTD while SNR increasing. The reason for this situation is that the interval among the velocities during phase compensation limits the estimation accuracy of the proposed method, and the speed interval during phase compensation can be reduced to further improve the estimation accuracy. From the above numerical simulations, we can see that the detection performance of proposed method is superior to that of the general MTD when SNR is lower than −20 dB, and their performance is similar on other SNRs.

## 5. Evaluation on Measured Data

### 5.1. Ground Vehicle Detection

**Figure 5:**
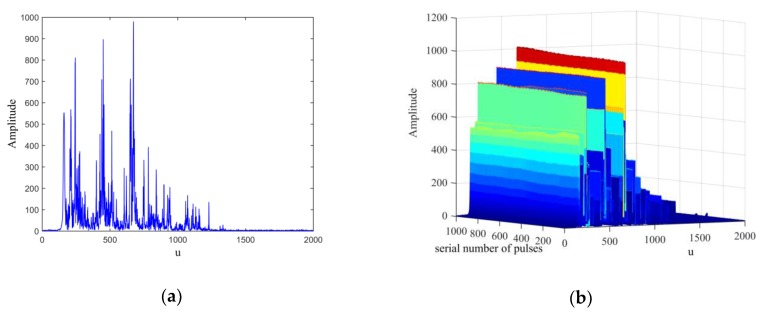
Fractional Fourier transform (FRFT) results of measured echoes with pulse repetition interval $T_{pri}=200\;\mu s$ and sampling frequency $f_{s}=10\;\mathrm{MHz}$ (**a**) FRFT of a single echo; (**b**) FRFT of 1024 continuous echoes.

To further verify the effectiveness of the proposed algorithm, we used data from an L-band radar test system to detect vehicles on roads, thereby demonstrating the applicability of the proposed algorithm to the detection of ground vehicles. The radar parameters include center frequency $f_{c}=1330\;\mathrm{MHz}$, pulse width $T_{p}=8\;\mu s$, bandwidth B=8 MHz, pulse repetition interval $T_{pri}=200\;\mu s$, sampling frequency $f_{s}=10\;\mathrm{MHz}$, pulse echo sampling length $N_{s}=2000$, and target distance of approximately 3 km. The proposed algorithm and general MTD algorithm were used to process the measured data. First, according to the radar parameters, we conducted data simulations to replicate the radar reception of the echo. According to Equation (16), we calculated the best rotation angle, $\alpha_{0}$ of the FRFT, and then applied the transform to *M* = 1024 echoes. Assuming that the maximum operating speed of the vehicles was 50 m/s, the speed interval for phase compensation was set to 0.5 m/s, and the FRFT of the echoes was divided into the positive and negative directions. From Equation (16), the optimal rotation angle $\alpha_{o}=0.0159$ of the FRFT can be obtained. The FRFT results of all measured echoes are shown in Figure 4. Figure 5a shows that the measured echo contains several LFM signal components with many peaks in the FRFT domain. Moreover, the FRFT result of 1024 continuous echoes in Figure 5b shows that the peak amplitude of the desired target in the fractional domain is very small, and the echo of the moving target is mixed with several ground clutter signals. Hence, it is difficult to detect the moving target only from the peaks in the fractional domain.

**Figure 6:**
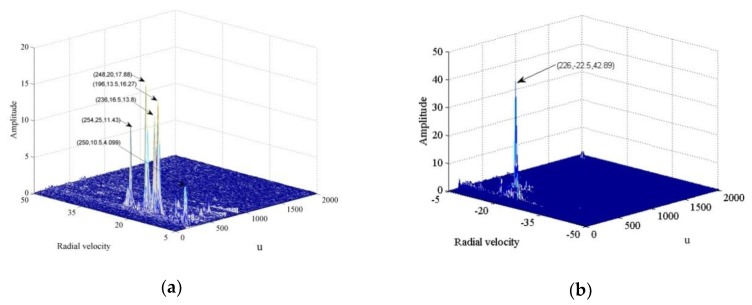
Proposed algorithm applied to ground vehicle detection and speed estimation. (**a**) Five targets with different velocities in the positive speed direction; (**b**) one target with velocity −22.5 m/s in the negative speed direction.

The results of applying the proposed algorithm on the measured data are shown in Figure 6a. Phase compensation and coherent accumulation of positive velocity retrieve five effective peaks, with coordinates (250, 10.5, 4.099), (254, 25, 11.43), (236, 16.5, 13.8), (196, 13.5, 16.27), and (248, 20, 17.88) for respective speed estimates of 10.5, 25.0, 16.5, 13.5, and 20.0 m/s. As shown in Figure 6b, the phase compensation and coherent accumulation of negative velocity yields one effective peak with coordinates (226, −22.5, 42.89) at an estimated speed of −22.5 m/s.

**Figure 7:**
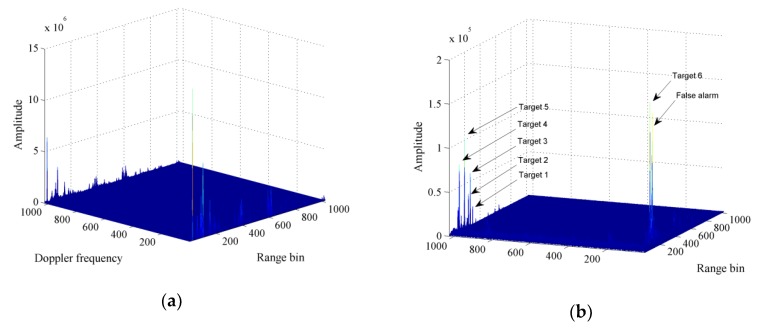
Moving target detection (MTD) algorithm applied to ground vehicle detection. (**a**) Range-Doppler plane without cancelling fixed objects; (**b**) range-Doppler plane with six true targets and a false alarm after eliminating zero frequency and four adjacent frequencies.

The results of applying the general MTD algorithm are shown in Figure 7. Figure 7a shows the range-Doppler plane obtained after applying the algorithm, where strong ground clutter appears at zero frequency, hindering the detection of the small and slow targets. Figure 7b shows the range-Doppler plane obtained after eliminating the peak at zero frequency and three close frequencies. Six effective targets, with one false positive, are detected. The false positive is located near the zero frequency, close to the target echo, being difficult to eliminate.

### 5.2. In-Flight UAV Detection

**Figure 8:**
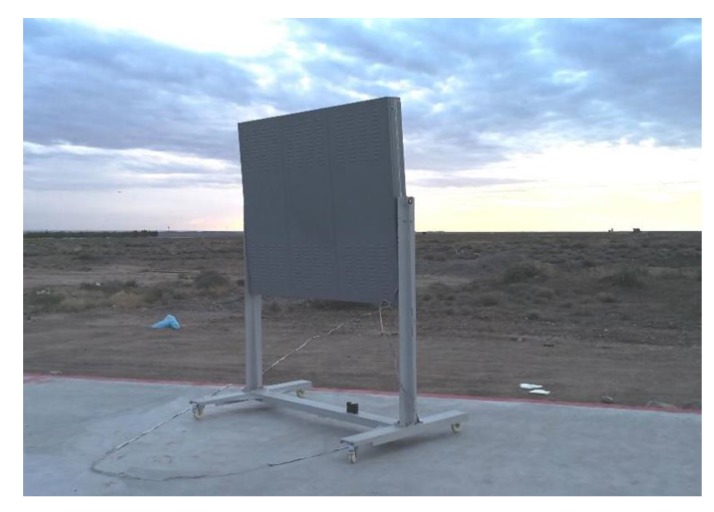
Picture of L-band array radar outfield.

**Figure 9:**
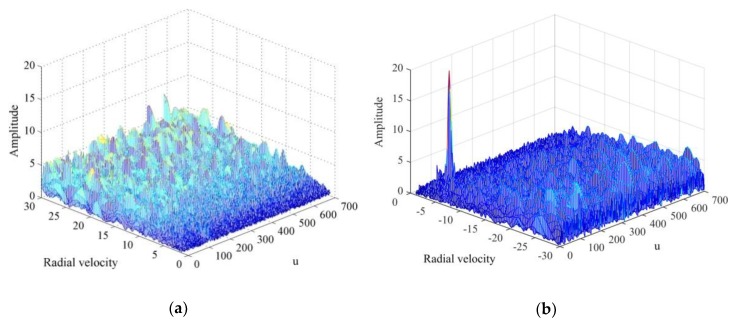
Proposed algorithm applied to UAV detection and speed estimation. (**a**) No targets detected in the positive speed direction; (**b**) one target detected with velocity −4.5 m/s in the negative speed direction.

**Figure 10:**
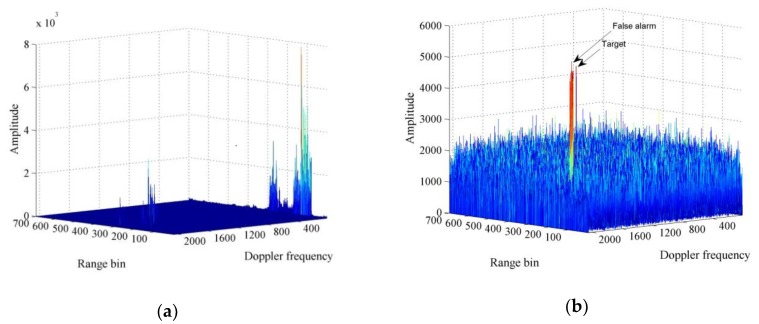
MTD algorithm applied to unmanned aerial vehicles (UAV) detection. (**a**) Range-Doppler plane without cancelling fixed objects; (**b**) range-Doppler plane with a true target and several false alarms after eliminating zero frequency and four adjacent frequencies.

We also evaluated the proposed algorithm on echoes obtained from a low-flying, small, and slow UAV acquired from another radar test system to detect targets on flat ground, as shown in Figure 8. The parameters of the radar system include center frequency $f_{c}=1330\;\mathrm{MHz}$, pulse width $T_{p}=8\;\mu s$, bandwidth B=2 MHz, pulse repetition interval $t_{pri}=280\;\mu s$, sampling frequency $f_{s}=2.5\;\mathrm{MHz}$, and pulse echo sampling length $N_{s}=700$. During data acquisition, the UAV maintained a cruising speed of −4.5 m/s at an approximate distance of 1.5 km from the radar. Assuming that the maximum flying speed of the target was 30 m/s, and the speed interval for phase compensation was 0.1 m/s, we conducted phase compensation and coherent accumulation on the FRFT of the echo considering velocity in the positive and negative directions. The corresponding results are shown in Figure 9, and Figure 10 shows the range-Doppler plane obtained from the MTD algorithm.

Figure 9a shows that there is no peak resulting from phase compensation and coherent accumulation of the positive velocity, indicating the absence of targets in the positive velocity region. Figure 9b shows an effective peak from the negative velocity processing, indicating the presence of the UAV at coordinates (91, −4.6, 19.94) with an estimated velocity of −4.6 m/s. Figure 10a shows that the general MTD algorithm retrieves several peaks at zero frequency and close frequencies in the range-Doppler frequency plane. After the removal of zero frequency and echoes of three nearby frequencies, Figure 10b shows that two peaks remain; one corresponding to the target echo, and the other reflecting lake clutter, which is a false positive requiring further processing to be eliminated.

## 6. Conclusions

For detection and speed estimation of moving targets, such as low-flying/ground, small, and slow targets, we proposed an algorithm based on fractional-domain phase compensation and coherent accumulation. After applying the corresponding FRFT to the continuous echoes, the peaks in the fractional domain indicated the LFM components, and the interferences were eliminated by phase compensation and coherent accumulation, thereby substantially improving the SINR. Accordingly, the desired target was identified using CFAR detection, and its radial velocity could be estimated using the velocity coordinate corresponding to the peak position. The effectiveness of the proposed algorithm was demonstrated by data simulation and processing of real echoes acquired from ground vehicles and a UAV flying over a lake. The algorithm could effectively detect multiple targets and accurately estimate the radial velocity, and it exhibited lower false alarm probability and lesser computational complexity than that of the general MTD algorithm. Furthermore, the speed estimation accuracy could be improved by adjusting the speed interval of phase compensation. In addition, applying the proposed method to practical application and phase compensation under different scenarios will be studied in the future.
